# Stable thyroid function despite regular use of povidone-iodine throat spray for SARS-CoV-2 prophylaxis

**DOI:** 10.1080/07853890.2022.2108132

**Published:** 2022-11-18

**Authors:** Amy May Lin Quek, Mei Yen Ng, Ooiean Teng, Nicole-Ann Lim, Geelyn Jeng Lin Ng, Samantha Peiling Yang, Mikael Hartman, Paul Anantharajah Tambyah, Alex R. Cook, Raymond Chee Seong Seet

**Affiliations:** a Department of Medicine, Yong Loo Lin School of Medicine, National University of Singapore, Singapore, Singapore; b Division of Neurology, Department of Medicine, National University Hospital, Singapore, Singapore; c Division of Endocrinology, Department of Medicine, National University Hospital, Singapore, Singapore; d Department of Surgery, Yong Loo Lin School of Medicine, National University of Singapore, Singapore, Singapore; e Saw Swee Hock School of Public Health, National University of Singapore and National University Health System, Singapore, Singapore; f Division of Infectious Diseases, Department of Medicine, National University Hospital, Singapore, Singapore; g Infectious Diseases Translational Research Program, Yong Loo Lin School of Medicine, National University of Singapore, Singapore, Singapore; h Healthy Longevity Translational Research Program, Yong Loo Lin School of Medicine, National University of Singapore, Singapore, Singapore

**Keywords:** Povidone-iodine, SARS-CoV-2, thyroid function

## Abstract

**Background:**

It is unclear whether unintentional ingestion of povidone-iodine following its application to the oropharyngeal space could affect thyroid function.

**Objective:**

To examine thyroid function among individuals who regularly apply povidone-iodine throat spray for SARS-CoV-2 prophylaxis.

**Methods:**

We designed a case-control study to compare thyroid function among participants who received povidone-iodine throat spray three times a day for 42 days (‘cases’) and those who received vitamin C (‘controls’). Thyroid function was assessed by profiling serum TSH, free T3, and free T4; iodine status was estimated using serum thyroglobulin level, while infection status was determined by measuring anti-SARS-CoV-2 antibody against the nucleocapsid antigen. All measurements were performed in pairs, at baseline and 42 days later. Pre-post changes in thyroid function were compared between groups, before and after stratification according to baseline TSH quartiles.

**Results:**

A total of 177 men (117 cases and 60 controls) (mean age, 32.2 years) were included. Despite comparable demographics and clinical profiles, no clinically or statistically significant differences were observed in thyroid indices between ‘cases’ and ‘controls’ before and after stratification according to TSH quartiles. None of the participants developed symptomatic hypo- or hyperthyroidism throughout the study. *Post-hoc* analysis did not reveal differences in thyroid function according to infection status.

**Conclusions:**

Data from this study support the overall safety of povidone-iodine use in the oropharyngeal space for SARS-CoV-2 prophylaxis among individuals with normal thyroid function and subclinical thyroid disease.

## Introduction

Povidone-iodine is a broad-spectrum antiseptic that is used to disinfect the oropharyngeal space [1–3]. Guidelines from the American Dental Association recommend pre-operative mouthwash and gargle of povidone-iodine before all dental procedures [1], while the Respiratory Society of Japan advocates povidone-iodine mouthwash to prevent transmission of respiratory illnesses in the community and among hospitalized patients [2]. Interest in povidone-iodine surged during this pandemic following preclinical reports showing SARS-CoV-2 virus inactivation following exposure to povidone-iodine [3–5] and data from clinical studies demonstrating a reduction in viral load among infected patients following oral gargle with povidone-iodine [3,6–9]. In a high transmission setting, a randomized controlled clinical trial that was carried out during an active outbreak of SARS-CoV-2 infection recorded a 24% absolute risk reduction in infection rates among those who regularly used povidone-iodine throat spray three times daily compared with an active comparator group that received vitamin C [10]. Whilst these data support providone-iodine use as a prophylaxis against SARS-CoV-2 infection, it is unclear whether frequent use of the throat spray for prolonged periods could affect thyroid function.

Iodine is a micronutrient that is required for the synthesis of thyroid hormones [11]. Although ingestion of iodine above the recommended daily intake of 150 µg in adults is generally well-tolerated [11], the impact of regular and prolonged use of povidone-iodine on thyroid function has not been rigorously studied. For example, overt hypothyroidism was reported in an elderly man who gargled undiluted povidone-iodine (7% concentration) daily for more than 10 years [12], but smaller cohort studies have observed a mild and subclinical increase in thyroid-stimulating hormone (TSH) after daily use of povidone-iodine mouth rinse for 6 months as part of gingivitis and dental plaque management [13], and in the treatment of recalcitrant chronic rhinosinusitis [14], without significant changes in triiodothyronine (T3) and thyroxine (T4) levels [13]. Among 12 hospitalized patients with SARS-CoV-2 infection, daily use of povidone-iodine four times a day for 5 days was found to increase TSH levels in ∼40% of subjects before normalizing 7–12 days later; no significant changes were observed in T3 and T4 levels [15]. Additionally, it is unclear whether those with subclinical thyroid disease could be more predisposed to changes in thyroid hormones and whether prospective users of topical povidone-iodine prophylaxis should be screened for thyroid disease before regular application. Previous attempts at examining the long-term effects of povidone-iodine were limited by a small sample size, incomplete biochemical assessment of thyroid function, a lack of comparison with suitable controls, and uncertainties surrounding the compliance of participants to the treatment protocol.

To examine the impact of frequent and repeated use of povidone-iodine throat spray on thyroid function, we employed the resources of the DORM trial [10] and designed a nested case-control study to compare the thyroid function of individuals who received povidone-iodine throat spray three times daily for 42 days with a control group comprising individuals who received oral vitamin C. We hypothesize that an increase in circulatory iodine (if any) following regular use of povidone-iodine in the oropharyngeal space is likely mild and is insufficient to affect thyroid function.

## Methods

### Study participants

All participants were men, aged between 21 and 60 years, who were recruited for the DORM trial during an outbreak of SARS-CoV-2 infection involving migrant workers in Singapore [10,16]. We included asymptomatic individuals, those between 21 and 60 years, and those who were willing to provide written informed consent, but excluded individuals with acute respiratory symptoms (either fever, runny nose, cough, and/or shortness of breath), dysgeusia or anosmia, and symptomatic thyroid disease.

‘Cases’ were identified from participants randomized to receive povidone-iodine throat spray three times daily for 42 days who reported full compliance with the treatment protocol. At study entry, participants in this group were shown and taught the proper use of the throat spray before being dispensed a 50 ml bottle of povidone-iodine 0.45% (Betadine, Mundipharma Pte Ltd). Compliance was assessed by examining self-reported compliance data captured using an encrypted mobile application (FormSG) developed by Singapore’s GovTech Data Science and Artificial Intelligence Capability Centre, and by measuring the residual volume of povidone-iodine at the end of the 42-day study. A participant was considered to be fully compliant if they did not miss any treatment dose and returned a bottle with <40% of the initial volume. ‘Controls’ were selected from age-matched participants who were randomized to receive an active comparator (oral vitamin C 500 mg/day, Blackmore Pte Ltd) but recorded a compliance rate of <30% which was verified by counting the number of unconsumed pills at week 6; the latter condition was added to derive expected thyroid function values that are closer to placebo estimates. The proportion of individuals with infection in both groups at baseline and on day 42 was balanced to account for possible confounding effects of acute infection on thyroid function. Blood pressure (mmHg) and heart rate (per min) were measured on recruitment. Body mass index (BMI) was calculated by dividing weight in kilograms by the square of height in meters. The trial was approved by the Domain-Specific Review Board, National Healthcare Group (2020/00561), the Ministry of Health, the multi-ministerial Joint Task Force, and was conducted under a Clinical Trial Authorization (CTA2000053) by the Health Sciences Authority which oversees all clinical trials in Singapore.

### Laboratory analyses

Following absorption, iodine is metabolized to form thyroid hormones and excess iodine is stored as thyroglobulin [17–19]. Serum TSH, free T3, and free T4 were measured to assess thyroid hormonal status, whilst thyroglobulin was measured to detect excess circulatory iodine (Cobas e411, Roche, Germany) [17–19]. Iodine was not measured directly using inductively coupled plasma mass spectrometry for cost considerations. All participants were assessed for anti-SARS-CoV-2 antibodies against the nucleocapsid antigen at baseline and on day 42 (Cobas e411, Roche, Germany).

### Statistical analyses

Categorical variables are summarized as numbers (*n*) and percentages (%). Continuous variables are expressed as mean (standard deviation) or median (interquartile range, IQR), and, based on their distribution, compared using the Student’s *t*-test and Mann–Whitney *U* tests as appropriate. Pre-post changes in individual thyroid indices were compared using the Mann–Whitney *U* test at baseline and on day 42, and in the adjusted analysis, through linear regression of the logged biomarkers or the logged ratio of final to baseline measurements. To examine whether individuals with subclinical thyroid disease could be more prone to changes in thyroid function, participants were categorized into quartiles based on their baseline TSH values. Changes in thyroid indices were compared between cases and controls within each quartile. To evaluate the impact of SARS-CoV-2 infection on thyroid function, participants were categorized into different seroconversion groups based on their baseline and day 42 serology status (negative to negative, negative to positive, and positive to positive); pre-post changes in thyroid indices were compared between these groups. Sample size calculation was not performed *a priori* and was based on pragmatic considerations, such as sample availability and funding. *Post-hoc* assessment of sample size demonstrated 75% power to detect a 20% difference in the geometric mean change in all four biomarkers over the study duration, which was an effect size considered to be clinically meaningful. SPSS Statistics version 27 (IBM Corporation, Armonk, NY, USA) and R 4.1.1 [20] were used for all analyses.

## Results

**Figure 1.**
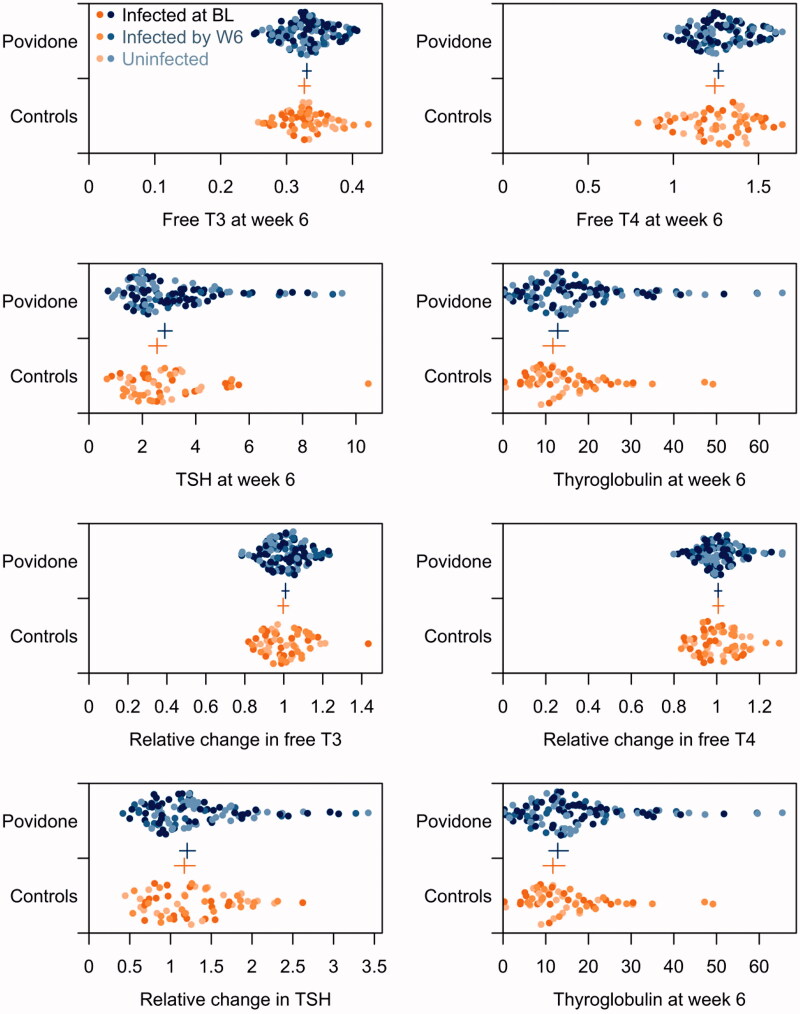
Distribution of biochemical indices of thyroid function. Dots represent empirical data; cross represents geometric mean and 95% confidence interval. Dots are colored by case (blue) or control (orange) and shaded by infection status at baseline (BL) and by week 6 (W6). Relative change is the ratio of W6 to BL biomarker. T3: triiodothyronine; T4: thyroxine; TSH: thyroid stimulating hormone.

A total of 177 men (117 cases and 60 controls) (mean age, 32.2 years) were included in this study. Cases and controls were comparable in terms of demographics, risk factors, clinical parameters, and SARS-CoV-2 infection status (Table 1). The prevalence of subclinical hypothyroidism in the cohort was 6.8% (defined as TSH >4.50 mIU/l) which was comparable between cases and controls (*n* = 7 and *n* = 5, respectively). Despite regular use of povidone-iodine, no clinically and statistically significant differences were observed in thyroglobulin and in the pre-post changes in thyroglobulin between cases and controls (Table 2, Figure 1). None of the participants had complained of pain overlying their thyroid gland or developed features of hypo- or hyperthyroidism throughout the study. Similarly, no clinically or statistically significant differences were observed in free T3, free T4, and TSH between cases and controls; neither were there differences in pre-post changes between groups (Table 2, Figure 1). Confidence intervals for the geometric mean values, and for the relative change over the study, of free T3, free T4, TSH, and thyroglobulin were narrow, yet overlapping between cases and controls, and the maximum plausible difference between the two groups was not clinically meaningful for any of the four biomarkers (Figure 1). No clinically or statistically significant differences in free T3, free 4, TSH, and thyroglobulin between seroconversion groups were observed (Table 3).

**Table 1.** Details of study participants.

	Cases (*n* = 117)	Control (*n* = 60)	*p*
Demographic			
Age (years)	32.0 (6.7)	32.7 (7.8)	0.61
Nationality (%)			0.24
Bangladeshi	26 (22%)	17 (28%)	
Indian	91 (78%)	42 (70%)	
Others	0	1 (2%)	
Risk factors (%)			
Hypertension	1 (0.9%)	0	1.0
Hyperlipidemia	0	0	1.0
Diabetes mellitus	0	0	1.0
Clinical parameters			
Systolic blood pressure (mmHg)	132 (14)	131 (21)	0.84
Diastolic blood pressure (mmHg)	85 (11)	86 (12)	0.75
Pulse rate (per min)	93 (13)	94 (15)	0.64
Body mass index (kg/m^2^)	24 (3)	24 (4)	0.69
SARS-CoV-2 seropositivity (%)			0.91
Baseline	38 (33%)	20 (33%)	
Day 42	78 (67%)	40 (67%)	

Data are summarized as mean (standard deviation) or *n* (%).

**Table 2.** Comparison in thyroid indices between cases and controls.

	Cases (*n* = 117)	Controls (*n* = 60)	*p*	Adjusted geometric mean ratio (95%CI)	*p*
Baseline					
Free T3 (ng/dl)	0.3 (0.04)	0.3 (0.04)	0.94	1.00 (0.96–1.04)	0.98
Free T4 (ng/dl)	1.3 (0.2)	1.3 (0.2)	0.51	1.02 (0.98–1.06)	0.41
TSH (mIU/l)	2.3 (1.8–3.2)	2.1 (1.6–2.9)	0.22	1.08 (0.94–1.24)	0.27
Thyroglobulin (ng/ml)	14.4 (9.3–20.0)	11.7 (7.8–19.6)	0.27	1.08 (0.81–1.44)	0.60
Day 42					
Free T3 (ng/dl)	0.3 (0.04)	0.3 (0.03)	0.44	1.01 (0.98–1.05)	0.50
Free T4 (ng/dl)	1.3 (0.2)	1.3 (0.2)	0.57	1.02 (0.97–1.06)	0.44
TSH (mIU/l)	2.7 (2.0–4.0)	2.6 (1.9–3.6)	0.28	1.11 (0.95–1.30)	0.18
Thyroglobulin (ng/ml)	14.2 (8.9–22.1)	12.2 (8.5–19.4)	0.24	1.09 (0.82–1.46)	0.54
Pre-post changes					
Free T3 (ng/dl)	0.01 (–0.02–0.02)	–0.00 (–0.03–0.02)	0.27	1.01 (0.98–1.04)	0.45
Free T4 (ng/dl)	0.0 (–0.1–0.7)	0.0 (–0.1–0.1)	0.84	1.00 (0.97–1.03)	0.96
TSH (mIU/l)	0.4 (–0.3–1.4)	0.3 (–0.4–1.2)	0.66	1.03 (0.90–1.18)	0.66
Thyroglobulin (ng/ml)	0.1 (–2.1–3.4)	0.5 (–0.9–2.4)	0.72	1.01 (0.92–1.11)	0.77

T3: triiodothyronine; T4: thyroxine; TSH: thyroid stimulating hormone.

Data are summarized as mean (standard deviation) or median (interquartile range). *p*-Values were calculated using student *t*-test for variables represented as means and *SD*s (and in the geometric means) and the Mann–Whitney *U* test for variables represented as medians and IQRs. Geometric mean ratios are adjusted for SARS-CoV-2 infection status at baseline and follow-up.

**Table 3.** Comparison in thyroid indices according to SARS-CoV-2 status at baseline and on day 42.

	Negative to negative (*n* = 59)	Negative to positive (*n* = 60)	Positive to positive (*n* = 58)	*p*
Pre-post changes				
Free T3 (ng/dl)	0.0 (–0.28–0.02)	0.0 (–0.02–0.02)	0.01 (–0.02–0.03)	0.64
Free T4 (ng/dl)	0.03 (–0.06–0.09)	0.01 (–0.06–0.09)	–0.02 (–0.09–0.07)	0.25
TSH (mIU/l)	0.28 (–0.38–1.26)	0.37 (–0.42–1.14)	0.47 (–0.30–1.48)	0.62
Thyroglobulin (ng/ml)	0.13 (–1.41–3.02)	–0.12 (–2.02–2.53)	0.52 (–0.89–2.54)	0.50

T3: triiodothyronine; T4: thyroxine; TSH: thyroid stimulating hormone.

Data are summarized as median (interquartile range). *p*-Values were adjusted for intervention.

## Discussion

Findings from this study suggest that regular use of povidone-iodine for prolonged periods is not associated with thyroid dysfunction among individuals with normal thyroid function, nor in those with subclinical thyroid disease. No significant differences in thyroid function were observed between individuals according to their SARS-CoV-2 infection status. To our knowledge, this study is the first to rigorously examine the impact of povidone-iodine throat spray by measuring different indices of thyroid function and included individuals who were highly compliant with the treatment protocol and a control arm that would allow the detection of minor changes in thyroid function but within normal limits.

**Figure 2.**
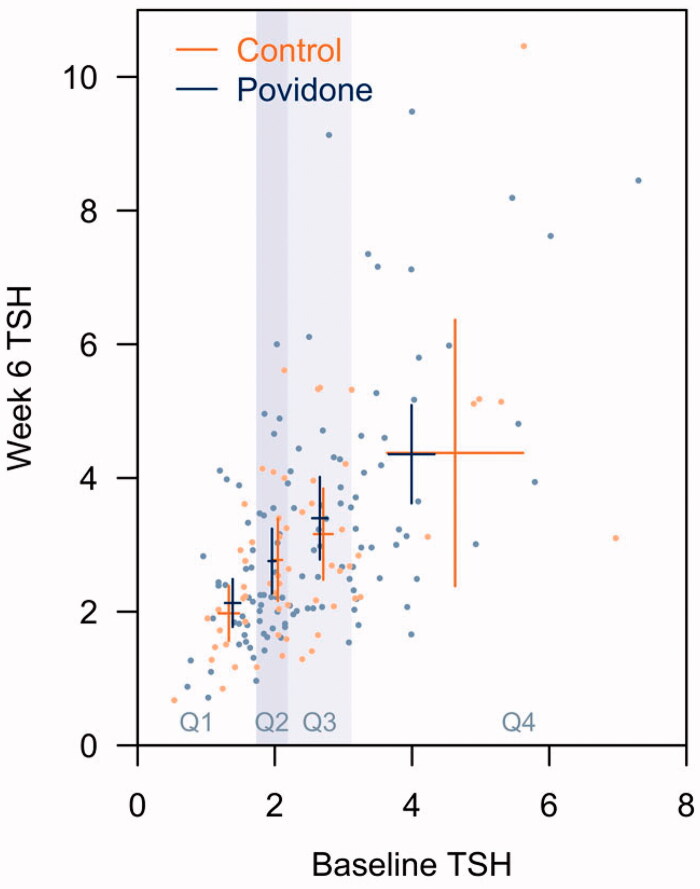
TSH measurements at baseline and follow-up by treatment. Points represent individual values, crosses represent 95% confidence intervals within quartiles at baseline. Shaded areas demarcate quartiles.

Our findings are consistent with one study that observed no change in thyroxine levels among 24 men following irrigation of the oral cavity with povidone-iodine compared with those who irrigated with chlorhexidine gluconate [21]. The lack of change in thyroid function suggests that any increase in circulatory iodine is likely compensated by the autoregulatory function known as the Wolff-Chaikoff effect which prevents thyroid tissues from synthesizing thyroid hormones despite the increased availability of iodine [22]. Although most povidone-iodine product labels caution against its use among individuals with thyroid disease, we did not observe differences in thyroid function between cases and controls in the lowest and highest TSH quartiles (Figure 2). Data from the current study support the overall safety of povidone-iodine use among those with subclinical thyroid disease which could argue against the need to screen prospective users for asymptomatic thyroid disease. Further studies are needed to examine the effect of povidone-iodine on thyroid function among those with symptomatic thyroid disease.

The SARS-CoV-2 virus enters cells using the angiotensin-converting enzyme type 2 (ACE2) receptor. This receptor is also highly expressed in thyroid glands, at levels greater than those present in lung tissues [23,24]. Among infected patients, serum ACE2 levels have been shown to correlate closely with circulatory T3 and T4 levels [25]. Despite these preliminary data linking SARS-CoV-2 and its biological propensity to infect thyroid tissues, we did not observe significant differences in thyroid function between infected and non-infected controls, and the following seroconversion. These findings are consistent with a previous report that observed no change in thyroid function during convalescence [26] but differ from studies that observed significant differences in thyroid parameters during acute infection [27], although it remains unclear whether these changes occur as a result of a nonthyroidal illness syndrome or direct infection of thyroid tissues. Compared to autopsy findings in patients with SARS-CoV infection [28], existing observations suggest either mild or no damage to thyroid follicles among those with SARS-CoV-2 infection [29–32]. These findings suggest that SARS-CoV-2 could have a less pronounced effect on thyroid tissues, contrary to its biological predisposition to infection given the abundance of ACE2 receptors.

Several limitations merit mention. First, the study included relatively young and healthy men but did not involve older individuals with various comorbidities which may affect iodine absorption and excretion. Second, although serum thyroglobulin did not differ between cases and controls, we are unable to definitively conclude that regular use of povidone-iodine did not result in iodine absorption as we did not directly measure levels of circulatory iodine. Third, we did not include those with symptomatic thyroid disease to conclude on the safety or harm associated with povidone-iodine use in these individuals. Data from this study support the overall safety of povidone-iodine throat spray for SARS-CoV-2 prophylaxis among individuals with normal thyroid function and those with subclinical thyroid disease.

## Consent

This study is a nested case-control study involving data from participants in the DORM trial. Consent was obtained before the participants entered this trial. The trial was approved by the Domain-Specific Review Board, National Healthcare Group (2020/00561), the Ministry of Health, the multi-ministerial Joint Task Force, and was conducted under a Clinical Trial Authorization (CTA2000053) by the Health Sciences Authority which oversees all clinical trials in Singapore.

## Data Availability

The data reported in this study are available on request from the corresponding author, RSCS.
